# Supplementary material for: The urea-creatinine ratio on the seventh day predicts the short-term prognosis of spontaneous intracerebral hemorrhage: a retrospective study
Source: PeerJ. 2025 Aug 18;13:e19874. doi: 10.7717/peerj.19874 (PMC12369599; doi:10.7717/peerj.19874)
Supplement: Supplemental Information 4 [file peerj-13-19874-s004.docx]

Gender: 1: Male; 2: Female

Bleeding_site: 1: Supratentorial hemorrhage; 2: Infratentorial hemorrhage

Intraventricular_extension: 1: No; 2: Yes

Hematoma_shape: 1: regular; 2: irregular

BCR_group(30.68): 1: BCR≤30.68; 2: BCR＞30.68

MRS_group: 1: 0-2points; 2: 3-6 points
