# Supplementary material for: The urea-creatinine ratio on the seventh day predicts the short-term prognosis of spontaneous intracerebral hemorrhage: a retrospective study
Source: PeerJ. 2025 Aug 18;13:e19874. doi: 10.7717/peerj.19874 (PMC12369599; doi:10.7717/peerj.19874)
Supplement: Supplemental Information 5 [file peerj-13-19874-s005.doc]

mRS_group: Grouping based on MRS

GCS: Glasgow Coma Scale

WBC: White Blood Cell

HB: Hemoglobin

PLT: Platelet

NLR: Neutrophil-to-Lymphocyte Ratio

PT: Prothrombin Time

APTT: Activated Partial Thromboplastin Time

FIB: Fibrinogen

ALB: Albumin

ALT: Alanine Aminotransferase

AST: Aspartate Aminotransferase

Ca: Calcium

K: Potassium

Na: Sodium

BCR_day1: The blood urea nitrogen to creatinine ratio on the first day of admission

BCR_day7: The blood urea nitrogen to creatinine ratio on the 7th day of admission

BCR_group(30.68): Grouping by blood urea nitrogen to creatinine ratio (30.68)
